# Interactions between FGF23 and vitamin D

**DOI:** 10.1530/EC-22-0239

**Published:** 2022-09-26

**Authors:** Mohammed S Razzaque

**Affiliations:** 1 Department of Pathology, Lake Erie College of Osteopathic Medicine, Erie, Pennsylvania, USA

**Keywords:** vitamin D, 1α(OH)ase, FGF23, Klotho, PTH

## Abstract

Fibroblast growth factor‐23 (FGF23) controls the homeostasis of both phosphate and vitamin D. Bone-derived FGF23 can suppress the transcription of 1α‐hydroxylase (1α(OH)ase) to reduce renal activation of vitamin D (1,25(OH)_2_D_3_). FGF23 can also activate the transcription of 24‐hydroxylase to enhance the renal degradation process of vitamin D. There is a counter-regulation for FGF23 and vitamin D; 1,25(OH)_2_D_3_ induces the skeletal synthesis and the release of FGF23, while FGF23 can suppress the production of 1,25(OH)_2_D_3_ by inhibiting 1α(OH)ase synthesis. Genetically ablating FGF23 activities in mice resulted in higher levels of renal 1α(OH)ase, which is also reflected in an increased level of serum 1,25(OH)_2_D_3_, while genetically ablating 1α(OH)ase activities in mice reduced the serum levels of FGF23. Similar feedback control of FGF23 and vitamin D is also detected in various human diseases. Further studies are required to understand the subcellular molecular regulation of FGF23 and vitamin D in health and disease.

## Vitamin D metabolism

**Figure 1:**
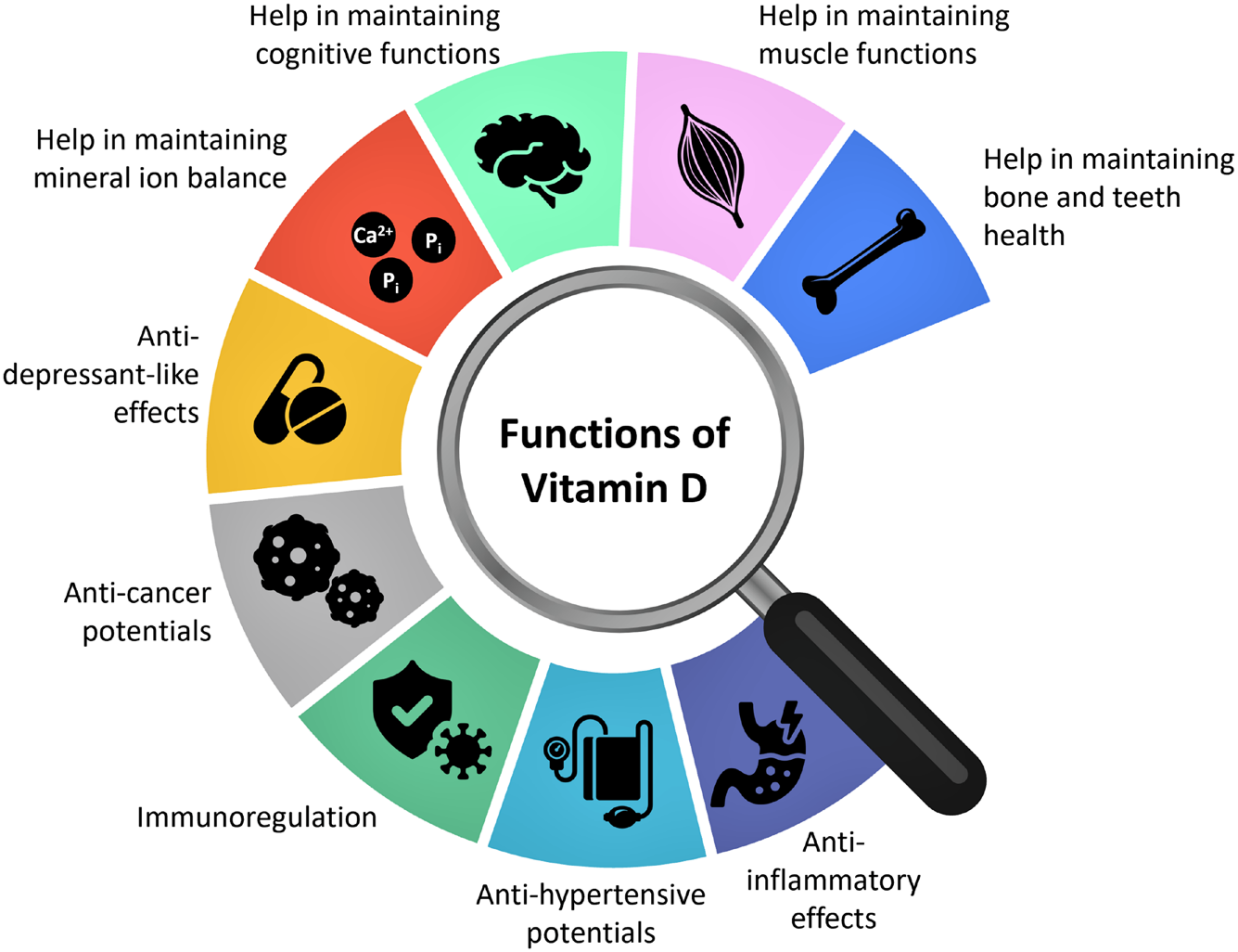
Simplified diagram illustrating various functions of vitamin D. Vitamin D is a multifunctional hormone and has been shown to have beneficial effects on the musculoskeletal system, the neuronal system, the immune system, and inflammatory pathways (1, 4, 34, 62, 63, 64, 65, 66).

Vitamin D regulates mineral ion homeostasis and skeletogenesis (1). The synthesis process of vitamin D initiates in the skin and is processed further in the liver and kidney to generate bioactive vitamin D. The biologically functional metabolite, 1,25 dihydroxy vitamin D3 (1,25(OH)_2_D_3_), is generated by two successive hydroxylations in the liver by 25 hydroxylase (CYP27A1) and in the kidney by 1α-hydroxylase (1α(OH)ase; CYP27B1). When 1,25(OH)_2_D_3_ level reaches optimal, the 24-hydroxylase (CYP24) catabolizes vitamin D, mainly in the kidney. The homeostatic control of vitamin D is partly regulated by the negative feedback of 1,25(OH)_2_D_3_ through suppressing the renal expression of 1α(OH)ase and stimulating the renal expression of 24-hydroxylase (2). The bioactive 1,25(OH)_2_D_3_ interacts with the high-affinity vitamin D receptor (VDR) to exert its functions (3). VDR forms a heterodimer with the retinoid receptor to induce the transcription of vitamin D-dependent genes by interacting with vitamin D-responsive elements (VDREs) in the promoter region of target genes (3). Several important mineralization-promoting genes are regulated by 1,25(OH)_2_D_3_ (4). Whether 1,25(OH)_2_D_3_ can directly influence the skeletal mineralization process is unclear. An exogenous infusion of calcium and phosphate to the severe vitamin D-deficient rats resulted in skeletal mineralization, similar to the group that received only vitamin D (5). Likewise, the rickets phenotype of genetically modified vitamin D-deficient mice could be rescued by providing adequate calcium and phosphate (6). However, a recent case report of rickets and hypophosphatasia in an infant girl with normal serum calcium and phosphate levels showed remarkable improvements in skeletal mineralization following vitamin D treatment, suggesting the possibility of a direct effect of vitamin D on the mineralization process (Fig. 1) (7). Several factors, including phosphate-regulating fibroblast growth factor‐23 (FGF23), can influence vitamin D metabolism by suppressing 1α(OH)ase activity (8).

## FGF23

FGF23 is a member of the endocrine FGF family, along with FGF19 and FGF21 (9). The biologically active form of intact FGF23 contains 227 amino acids. FGF23 is mainly synthesized and released from osteoblasts and osteocytes (10). Bioactive FGF23 (32‐kDa), in the presence of αKlotho, can bind with the FGF receptors (FGFR) to exert downstream signaling events (11). Studies have found that FGF23 exerts most of its physiologic functions through binding with FGFR1c (11, 12). In renal proximal tubular epithelial cells, circulating FGF23 binds with the αKlotho/FGFR1c complex to suppress the activities of sodium-phosphate cotransporters (NaPi‐2a and NaPi‐2c) to increase urinary phosphate excretion. NaPi‐2a is responsible for the renal reabsorption of most of the filtered phosphate, and its expression is regulated by FGF23, parathyroid hormone (PTH), and dietary phosphate intake (13). About one-third of renal reabsorption is mediated by NaPi-2c and regulated by FGF23, metabolic acidosis, dietary magnesium, and phosphate (13). Genetically ablating FGF23 or its obligate cofactor, αKlotho, reduced urinary phosphate excretion due to enhanced renal reabsorption of phosphate by increased activities of NaPi‐2a (12, 14, 15, 16). Human studies have found that vitamin D supplementation or administration of calcitriol (1,25(OH)_2_D_3_) could increase urinary phosphate excretion, perhaps by inducing FGF23 secretion (17). Alongside phosphaturic functions, FGF23 is able to repress the transcription of 1α(OH)ase in proximal renal tubular epithelial cells to influence vitamin D metabolism.

## Vitamin D and skeletal FGF23 synthesis

**Figure 2:**
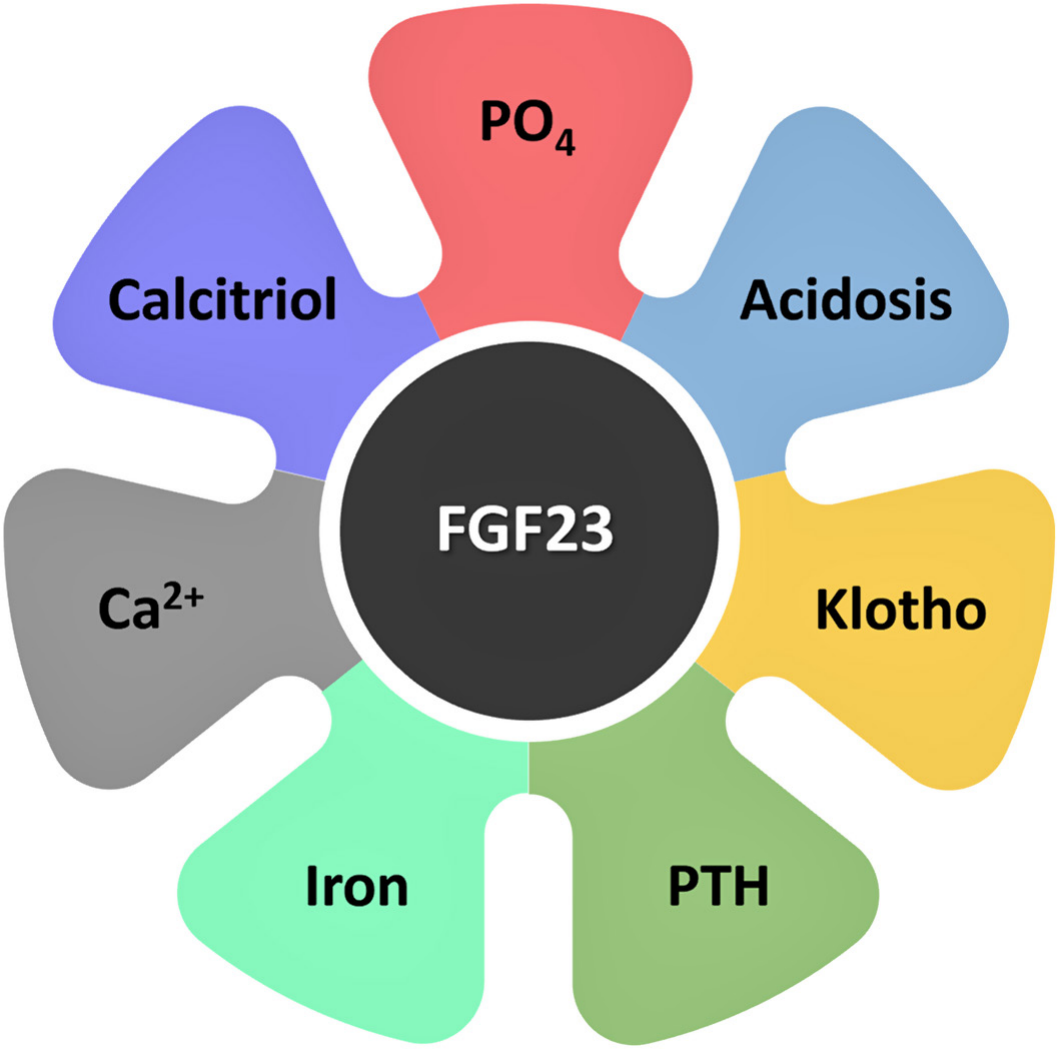
Simplified diagram illustrating various factors that can directly or indirectly influence FGF23 activities. Feedback control of FGF23 exists with phosphate and vitamin D. PO_4,_ phosphate; Ca^2+^, calcium (19, 67, 68, 69, 70, 71).

Skeletal secretion of FGF23 is regulated by local and systemic factors, including calcium, phosphate, vitamin D, PTH, leptin, iron, acidosis, and inflammatory cytokines (Fig. 2) (18, 19, 20). Experimental studies have shown that FGF23 can suppress PTH secretion (21), although the human relevance of this observation is not yet clear. PTH is also claimed to induce FGF23 synthesis (22, 23). When rat osteoblastic cells were treated with 1,25(OH)_2_D_3,_ enhanced production of FGF23 was noted (24, 25). However, when osteoblastic cells were exposed only to phosphate, such enhancement of FGF23 was no longer detected, implying that in the* in vitro* microenvironment, phosphate alone is not able to induce FGF23 production (24, 26, 27). Moreover, when using co-treatment with commonly used transcriptional and translational inhibitors studies, the induction of FGF23 by 1,25(OH)_2_D_3_ has shown to be regulated at the transcriptional level, perhaps involving the nuclear VDR (25). In relevance to this, genetically inactivating VDR from mice resulted in reduced circulatory levels of FGF23 compared to the wild-type (WT) control mice (28); 1,25(OH)_2_D_3_ challenged VDR null mice did not show any response to FGF23 production, implying that functionality of VDR is required for the skeletal synthesis of FGF23. In a similar observation, 1α(OH)ase knockout mice with a functioning VDR system also demonstrated low circulatory levels of FGF23, but when treated with 1,25(OH)_2_D_3_, the 1α(OH)ase knockout animals were able to enhance FGF23 expression, once again implying the essential role(s) of the VDR system in regulating FGF23 synthesis (29). Meta-analysis of randomized, placebo-controlled trials (RCTs) found that vitamin D administration was significantly associated with increased circulating levels of FGF23 in a dose-dependent manner (30), although a separate study reported no such association between vitamin D supplementation and FGF23 levels (31). The nature of vitamin D supplementation, hormonal (1,25(OH)_2_D_3_) vs nutritional (25(OH)D) forms, might partly explain such discrepancies. Of biological significance, the human FGF23 gene contains VDREs in its promoter region that can mediate 1,25(OH)_2_D_3_ and VDR actions (32). Furthermore, FGF23 influences the catabolism of 1,25(OH)_2_D_3_ into an inactive metabolite (33) to reduce the intestinal absorption of calcium and phosphate (34). Vitamin D supplementation has been shown to exert positive effects on the induction of αKlotho in chronic kidney disease (CKD) patients undergoing hemodialysis (35). Similar induction of αKlotho is also reported in vitamin D challenged mice (8). The skeletal induction of FGF23 and renal induction of αKlotho by vitamin D forms a kidney–bone axis.

## FGF23 and renal vitamin D synthesis

A counter-regulation exists between FGF23 and vitamin D activities (26). When* in vivo* FGF23 functions were blunted, 1α(OH)ase expression level was high in the kidneys of both Fgf23 knockout mice and αklotho knockout mice, resulting in increased serum levels of 1,25(OH)_2_D_3_ (15, 16, 36). Similarly, increased expression of 1α(OH)ase and elevated serum levels of 1,25(OH)_2_D_3_ were detected in Fgf23 and αklotho double knockout mice (14), suggesting that disrupting the FGF23 signaling cascade can enhance vitamin D activities. Moreover, genetically restoring the systemic effects of bio-active FGF23 in Fgf23 knockout mice reversed the hypervitaminosis D (from high serum 1,25(OH)_2_D_3_ levels to low serum levels) that was consistently noted in fgf23 knockout mice (37), again providing an* in vivo* evidence of FGF23 and vitamin D interactions. An increased serum 1,25(OH)_2_D_3_ level with hypercalcemia, hyperphosphatemia, and ectopic calcification are also noted in human diseases with inactivating mutations in FGF23 (38) and/or αKLOTHO (39) genes. Contrary to the loss of function of FGF23, genetically modified mice overexpressing fgf23 showed markedly reduced serum 1,25(OH)_2_D_3_ levels (37, 40). However, when FGF23 signaling was disrupted by selectively inactivating FGFR1 from proximal tubular epithelial cells, FGF23 lost its ability to suppress 1,25(OH)_2_D_3_ production in mice (41). Injecting exogenous bioactive FGF23 into normal WT mice has been shown to decrease renal 1α(OH)ase expression and enhance the expression of 24‐hydroxylase in the proximal tubular epithelial cells (42). When recombinant FGF23 was injected into the VDR‐knockout mice, it could suppress 1α(OH)ase similar to the WT mice but could not increase the expression of 24‐hydroxylase, suggesting the involvement of VDR in FGF23-mediated regulation of 24‐hydroxylase (43).

The Hyp mouse is the murine model of human X-linked hypophosphatemia (XLH), with a loss-of-function mutation in the PHEX gene. Hyp mice develop severe hypophosphatemia due to high circulating levels of bioactive FGF23 and activation of Erk1/2 signaling (44). It is believed that FGF23 and FGFR interactions activate the downstream MAP kinase signaling pathway to exert its bioactivities. Blunting the FGF23 activities by inhibiting Erk1/2 actions in Hyp mice resulted in an increase of 1,25(OH)_2_D_3_ levels (45), suggesting an* in vivo* interaction of FGF23-vitamin D. Similarly, when FGF23 bioactivities were blunted in Hyp mice by genetically ablating αklotho functions, increased serum levels of 1,25(OH)_2_D_3_ were detected (44). Although further studies are required to dissect exact molecular interactions, existing human and animal studies suggest that FGF23 is an endogenous regulator of vitamin D that can fine-tune the synthesis and functions of vitamin D (46, 47).

## Translational implications

Understanding the physiologic regulation and interaction of FGF23 and vitamin D helped in determining the pathomechanisms of renal phosphate-wasting diseases, including XLH, autosomal dominant hypophosphatemic rickets, or tumor-induced osteomalacia (TIO); all three of these diseases are characterized by extremely high circulating levels of bioactive (intact) FGF23 (48, 49). Furthermore, higher bioactive FGF23 in these diseases reduces the generation of 1,25(OH)_2_D_3_ to lower intestinal phosphate absorption. The cumulative effect of increased renal phosphate wasting and reduced intestinal phosphate uptake diminishes skeletal mineralization due to persistent hypophosphatemia (50). Therefore, therapeutically, decreasing the activities of FGF23 in patients with XLH improved phosphate balance and reduced skeletal defects (51). Burosumab, a human MAB, binds and blocks the action of FGF23. A double-blind, placebo-controlled, phase 3 trial with burosumab on symptomatic adults with XLH showed improvements in the mineralization process of preexisting unmineralized bone matrix (52). The safety profile of FDA-approved burosumab is claimed to be similar to placebo (53). Beneficial effects of reducing FGF23 activities by administering burosumab are also reported in a patient with TIO; burosumab normalized serum phosphate levels without phosphate supplementation within 2 months of treatment (54). Moreover, neutralizing the effects of FGF23 by burosumab normalized bone biomarkers and improved pseudofractures of the patient (54). In patients with cutaneous skeletal hypophosphatemia syndrome, where conventional treatment failed to achieve desired benefits, burosumab treatment can improve the clinical symptoms of hypophosphatemic rickets (55, 56). Of relevance, cutaneous skeletal hypophosphatemia syndrome is a rare illness caused by the gain-of-function mutations of RAS family gene, causing epidermal nevi, dysplastic cortical bony lesions, and FGF23-induced hypophosphatemic rickets (57). Ongoing clinical studies are suggesting therapeutic potentials of manipulating FGF23-vitamin D axis in patients with mineral ion dysregulation and skeletal deformities.

## Conclusion

**Figure 3:**
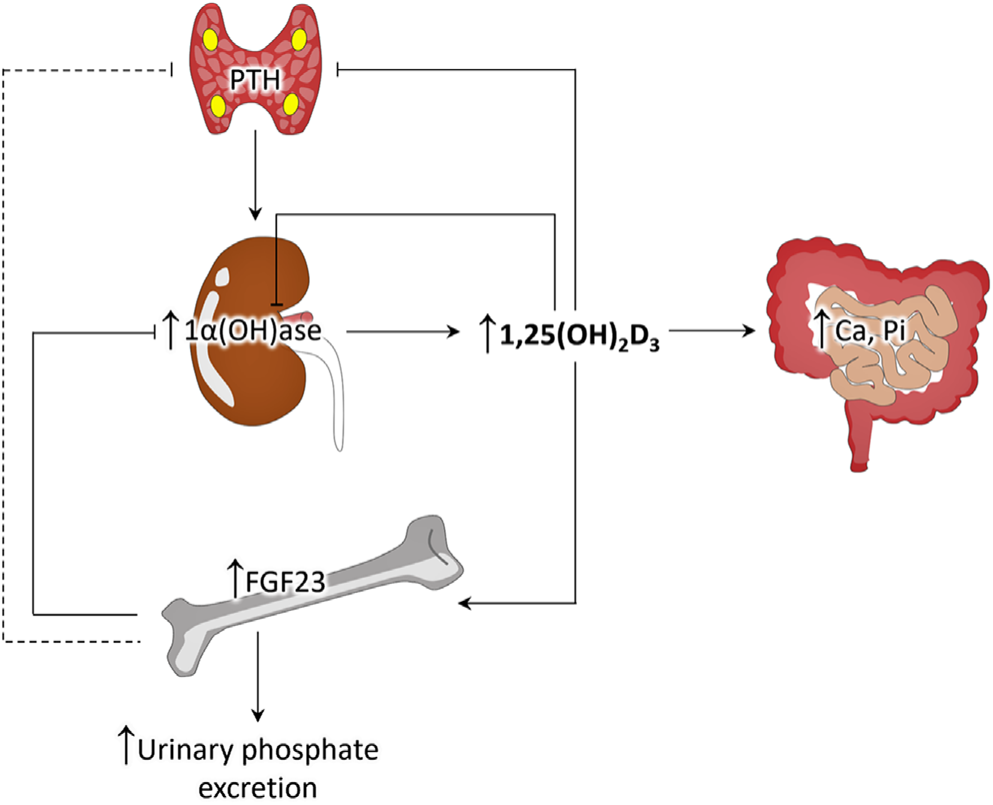
Simplified diagram illustrating vitamin D interactions with PTH, FGF23, calcium, and phosphate. PTH increases the synthesis of 1α(OH)ase and thereby increases 1,25(OH)_2_D_3_, which in turn exerts suppressive effects on PTH production. 1,25(OH)_2_D_3_ also enhances intestinal absorption of calcium and phosphate. 1,25(OH)_2_D_3_ can induce skeletal synthesis of FGF23, which exerts inhibitory effects on 1α(OH)ase to reduce 1,25(OH)_2_D_3_ levels. In addition, FGF23 increases urinary phosphate excretion and is likely to exert inhibitory effects on PTH. Moreover, 1,25(OH)_2_D_3_ can control its own balance by inhibiting the bioactivities of 1α(OH)ase. Ca, calcium; Pi, phosphorus.

I briefly highlighted the underlying mechanisms of FGF23 and vitamin D interactions. A counter-regulation between FGF23 and vitamin D synthesis appears to fine-tune the functions of both FGF23 and vitamin D. Bone and kidney cross-talk is actively regulating the FGF23-vitamin D axis. Kidney-derived active 1,25(OH)_2_D_3_ acts on the bone cells to produce FGF23, which in turn decreases renal 1,25(OH)_2_D_3_ synthesis (Fig. 3). Such reduction of 1,25(OH)_2_D_3_ is achieved by FGF23-induced downregulation of 1α(OH)ase expression in the kidney. In the reduced FGF23 microenvironment, the intrinsic regulation of 1,25(OH)_2_D_3_ is impaired, causing high circulating 1,25(OH)_2_D_3_ levels. In elderly individuals, serum FGF23 levels were relatively higher (58). Whether such elevation is related to generally low vitamin D levels in this group of people will require further study. Higher FGF23 level in patients with CKD is associated with reduced production of 1,25(OH)_2_D_3_, though part of it might be related to the reduced functional renal mass. Whether the conventional approach of using vitamin D analogs to treat patients with CKD could further enhance the levels of FGF23 to exacerbate non-renal adverse effects would need careful clinical consideration (59). Human studies have shown that cardiovascular and all-cause mortality risk rises gradually and progressively as the level of FGF23 increases (60, 61). Of biological importance, PTH‐mediated induction of 1α(OH)ase in the kidney is unable to compensate for the FGF23-mediated renal suppression of 1α(OH)ase, once more emphasizing the crucial involvement of FGF23 in vitamin D metabolism (Fig. 3). Finally, how subcellular signaling events regulate skeletal FGF23 synthesis and renal regulation of vitamin D activation and catabolism will need further study.

## Declaration of interest

The author declares that there is no conflict of interest that could be perceived as prejudicing the impartiality of this review.

## Funding

This work did not receive any specific grant from any funding agency in the public, commercial or not-for-profit sector.
